# Design of industrial wastewater demulsifier by HLD-NAC model

**DOI:** 10.1038/s41598-021-95485-7

**Published:** 2021-08-09

**Authors:** Hassan Ghasemi, Fatemeh Eslami

**Affiliations:** Department of Chemical Engineering, Tarbiat Modares University, Jalal Al Ahmad HWY, P.O. Box, 14115-111 Tehran, Iran

**Keywords:** Chemical engineering, Surfaces, interfaces and thin films

## Abstract

The chemical method is one of the treatment techniques for the separation of oil–water emulsion systems. The selection of appropriate demulsifiers for each emulsion system is the most challenging issue. Hydrophilic-lipophilic-deviation (HLD) is a powerful semi-empirical model, providing predictive tools to formulate the emulsion and microemulsion systems. This work aims to apply HLD to obtain an optimal condition for demulsification of oil-in-water emulsion system—real industrial wastewater—with different water in oil ratios (WOR). Therefore, the oil parameter of the contaminant oil and surfactant parameter for three types of commercial surfactants were calculated by performing salinity scans. Furthermore, the net-average-curvature (NAC) framework coupled with HLD was used to predict the phase behavior of the synthetic microemulsion systems, incorporating solubilization properties, the shape of droplets, and quality of optimum formulation. The geometrical sizes of non-spherical droplets (*L*_*d*_*, R*_*d*_)—as an indicator of how droplet sizes are changing with HLD—were consistent with the separation results. Correlating *L*_*d*_*/R*_*d*_ at phase transition points with bottle test results validates the hypothesis that NAC-predicted geometries and demulsification behavior are interconnected. Finally, the effect of sec-butanol was examined on both synthetic and real systems, providing reliable insights in terms of the effect of alcohol for WOR ≠ 1.

## Introduction

Nowadays, the negative impacts of oily wastewaters have been increasing, and because of their operational and environmental disadvantages, proper separation methods must be used to alleviate safety concerns^1–3^. The chemical method through using demulsifiers is widely recommended as the first choice for treating water-in-crude oil emulsions on industrial scales due to its higher performance than other methods^4^.

Salager’s semi-empirical correlation of hydrophilic-lipophilic-deviation (HLD) is used in order to obtain the optimal formulation of emulsion treatment^5^. This correlation specifies a linear relationship between variables influencing the phase behavior of surfactant-oil–water (SOW) systems^6^. It should be noted that the insights delivered by the HLD model are also applicable to the demulsification or stability of emulsified systems since emulsion systems can be described by the equilibrium phase behavior of the corresponding microemulsion systems^7^. Therefore, according to the literature, the HLD of SOW systems can be expressed^8,9^:

1 $$HLD=F(s)-k(EACN)-f(A)-{\alpha }_{t}(\Delta T)+Cc$$

where *S* is the salinity of the aqueous phase (g _NaCl_/100 mL), reflecting the influence of salinity on the water-surfactant interactions. *F(S)* is the function of salinity, being expressed as *Ln(S)* and *b(S)* for ionic and nonionic surfactants, respectively. Equivalent Alkane Carbon Number (*EACN*) represents oil hydrophobicity^10^ and *f(A)* is the function of alcohol, which usually acts as co-surfactant or co-solvent in the system^11^. It is expressed as *m*_*a*_*C*_*a*_ in the HLD formulation, where *m*_*a*_ is a constant value depending on the alcohol type and *C*_*a*_ represents its concentration^12^. $\Delta T$ is the temperature deviation from 25 °C, and *Cc* is the indicator of hydrophilicity of the surfactants in the system. *b, k* and ${\mathrm{\alpha }}_{\mathrm{t}}$ are constant parameters, depending on the nature of the system's materials^13,14^. According to HLD values, four types of phase behaviors named Winsor I, II, III, and IV are observable in SOW systems^15,16^. Winsor III of microemulsion systems is the optimal zone for demulsification, where the HLD value is nearly zero^17^. In addition, IFT and system stability reach their lowest values within Winsor III. It can be said that HLD plays a significant role in characterizing the phase behavior of SOW systems^15^. For example, the Phase Inversion Point (PIP), which happens at HLD = 0, provides important information for the formulation of SOW systems, such as addressing some issues about the self-assembly processes in pharmaceutical applications or choosing an appropriate demulsifier for a system of interest.

Although HLD is a well-known method to produce the desired types of SOW systems, it does not provide sufficient information about the physical properties of SOW systems. Moreover, the HLD model applies to WOR near 1. Therefore, Acosta and colleagues promoted the HLD concept by connecting it with a new complementary model termed net average curvature (NAC), thereby providing a tool capable of predicting SOW systems properties^18^. The NAC concept introduces two statistical explanations for the curvatures of surfactant film adsorbed at the interface. The first equation is^19^:

2 $${H}_{n}=\left(\frac{1}{{R}_{o}}-\frac{1}{{R}_{w}}\right)=\frac{-HLD}{L}$$

where *H*_*n*_ is the net curvature; *R*_*o*_ and *R*_*w*_ are the radii of coexisting hypothetical spherical droplets of oil and water, respectively. Details of the NAC calculation procedure depending on the Winsor type can be found elsewhere^20–22^. $L$ is a length scaling parameter that is the function of the surfactant tail chains tuned to the experimental results as a fitting parameter^15,22,23^.

The second equation regarding the HLD-NAC, which is called the average curvature, ${H}_{a}$, is represented by^18,19^:

3 $${H}_{a}=\frac{1}{2}\left(\frac{1}{{R}_{o}}+\frac{1}{{R}_{w}}\right)\ge \frac{1}{\xi }$$

It is generally accepted that the characteristic length parameter ($\xi$) can characterize the surfactant ability in solubilizing oil and water^24^. For instance, to predict the boundaries of three-phase regions where the radii of droplets cannot exceed the characteristic length, HLD-NAC can be implemented to identify the initial point of transition regions by defining the following equations^25^:

4 $${HLD}_{I-III}=\frac{2L}{{\mathrm{R}}_{\mathrm{w}}}-\frac{2L}{\xi }\approx -\frac{2L}{\xi }$$

5 $${HLD}_{II-III}=\frac{2L}{{\mathrm{R}}_{\mathrm{o}}}+\frac{2L}{\xi }\approx +\frac{2L}{\xi }$$

Here, similar to the *L* parameter, an experimental method of phase salinity scan is applied to obtain the characteristic length; because theoretical methods of calculating $\xi$ are not appropriate for all different classes of surfactants.

Based on the points mentioned above, it would seem that one of the most comprehensive ways to characterize the optimal zone for demulsification is by the HLD-NAC model. Here, we use two fundamental features of this model—which are closely interconnected—to evaluate the demulsification performance: solubilization properties and surfactant micellar structures. The former has been related to the phase volume fractions measurement and its connection with the quality of the optimum formulation, followed by calculation of *L* and $\xi$ through the salinity scans. The latter is associated with the elongation of micellar structures from the spherical shape (${L}_{d}$=0) to the cylindrical one (${L}_{d}\gg {R}_{d}$)^26^. The dimension of micellar structures ${L}_{d}$ and ${R}_{d}$ are predicted by two statistical explanations of HLD-NAC^7^.

Our goal is to apply the HLD-NAC method to demulsify the oil in water emulsion in the present study. In this way, exploring the PIP of microemulsion systems evaluates the demulsification performance effectively. Thus, we investigate two types of systems: industrial wastewater from an olefin plant named industrial emulsion *IE* and a synthesized oil in water microemulsion produced by the contaminant oil in *IE* called synthetic microemulsion system ($S\mu E$). This study has five objectives: (1) designing the fastest method to find the HLD parameters for the $S\mu E$ system. (2) examining the efficiency of the candidate surfactants based on the HLD concept by evaluating the phase separation process in $S\mu E$ and *IE.* (3) implementing the NAC model to examine the phase behavior of $S\mu E$ and validate the obtained results. (4) examining the sec-butanol effect on the separation process for both systems. Although several studies have shown alcohol effects on phase separation mechanism, our study is the first one reporting the alcohol effect on SOW systems with WOR $\ne 1$. (5) investigating the demulsifier concentration on the optimum condition.

## Materials and methods

### Chemicals

Both sodium dodecyl sulfate (SDS) and sodium bis-2-ethyl hexyl sulphosuccinate (AOT) surfactants (98%) were purchased from Sigma Aldrich, and Merck, respectively, and they were used as basis surfactants to measure the oil parameter. The surfactants Tween 80 (T80) (100%) and Span 20 (S20) (100%), as well as Hexane (99% +) as test oil, were supplied from Merck. The commercial surfactants KELA 3 (K3), KEOL 6 (K6), and Aria Kokoat (AK) were prepared from the Kimiagaran Company (Tehran, IR) to be used for wastewater treatment. The industrial wastewater (*IE*) and its contaminant oil used in this research were donated by Morvarid Petrochemical Company (Tehran, IR). Distilled water (< 5 $\mu s/cm$) and sec-butanol (99.5%) —which plays the role of solvent— were purchased from Merck.

### Determination of HLD parameters

As previously stated, *EACN,* the parameter identifying the power of hydrophobicity of the oils, is easily calculated through phase scans. In this research, the salinity scan was utilized to evaluate the *EACN*. Considering the low price and higher sensitivity of ionic surfactants to the salt compared with nonionic ones, these types of surfactants are mostly used for the phase scan^27^. To measure the *EACN* of the oil, we used NaCl and the mixture of SDS-AOT as the salt, and ionic surfactant, respectively. The properties of these surfactants are available in Supplementary Table S1. To reduce the number of experiments, specifying the scan’s scope before carrying out the salinity scan is recommended. Here, the initial guess for *EACN* was obtained through a GC/MS analysis (Supplementary Fig. S1) of the oil.

GC/MS analysis showed approximately 50 components in the oil. We classified it into two groups to make the initial guess for *EACN* possible. The components having definite *EACN*, such as linear alkanes, were placed in the first group and the rest in the second group. We estimated the EACN of the second group by assigning the *EACN* of the most similar oils to them structurally with well-defined values. Using the linear mixing rule based on weight fraction for the two groups, we reported 2.78 as the initial guess for the *EACN* of the oil. Finally, as Supplementary Fig. S2 shows, by performing the salinity scan, the three-phase system of Winsor III was observed in S = 2, which corresponded to the *EACN* of 1.7 for the contaminant oil (Supplementary Table S2).

In order to complete the HLD calculations, we needed the *Cc* of all the surfactants used in this study. The *Cc* parameters for Tween 80, Span 20, SDS, and AOT were found in the literature^28^. However, we performed the salinity scan to measure the *Cc* of the commercial nonionic surfactants such as K3, K6, and AK. To determine the optimal salinity of the scan, we carried out the same procedure as the *EACN* measurement: wherever the three-phase region was visible, the *Cc* value was identified (Supplementary Fig. S3). The *Cc* parameters are presented in Table 2. It should be noted that the calculation of EACN and *Cc* in the mixture of the oils or surfactants was based on the mass fraction linear mixing rule and mole fraction linear mixing rule, respectively^29,30^.

### Phase behavior scans of SOW systems with a mixture of surfactants

$S\mu E$ system was formulated with 5 mL of an aqueous phase consisting of a mixture of surfactants and 5 mL of the contaminant oil. In the aqueous phase, the surfactant concentration was constant and equal to 5 wt%. The salinity of the industrial wastewater was obtained 0.1 using a conductive detector. Due to this tiny quantity, we did not consider the salinity effect in $S\mu E$. Regarding the operating temperature of the working units in the wastewater treatment process in the related plants, the experiment is carried out at 25 °C ($\Delta T$ = 0). All vials containing microemulsions were shaken by hand 15–20 times and placed in suitable positions to reach an equilibrium state, taking 48 to 72 h. To investigate the efficiency of demulsifiers on phase separation in the real wastewater, we injected 10 mL of *IE* with WOR ≠ 1 into the tubes along with 5 wt% of surfactants.

### Determining the amount of phase separation

After reaching equilibrium, results were characterized by TOC (TOC-V CPN, Shimadzu) analysis to detect separation capability in both systems. 1 mL of the aqueous phase of any tube was poured into a special glass and placed at the TOC analyzer. The bottle test is also used in this study to monitor the volume of the separated phases in $\mathrm{S\mu E}$ system. Since the tubes are at equilibrium with the defined values, the efficiency of the mixtures of surfactants was simply calculated by dividing the separated volumes of oil by their initial volume in the systems.

### Effect of alcohol on phase separation

The effect of alcohol on the treatment process was examined by adding 2 volume percent of sec-butanol into the $S\mu E$ and *IE* systems.

### Effect of surfactant concentration on $\mathrm{S\mu E}$ phase behavior

0.5, 2, and 4 weight percent of the surfactant mixture of AK and K3 were prepared to be added in 10 mL vials of water (W) and oil (O). The aqueous phase containing surfactants and oil was gently mixed to reach phase equilibrium. After about 72 h, the results were examined.

## Results and discussion

The three surfactant mixtures were prepared to provide the desired *Cc* of the system for the demulsification purpose:

- Mixture (1): Tween80 (T80) + Span 20 (S20)
- Mixture (2): K3 + AK
- Mixture (3): K3 + K6

The same *Cc* values for these mixtures are set. Thus, using the mixing rule and their molecular weights, each mixture’s mole and weight fractions in both $S\mu E$ and *IE* were calculated easily.

### The efficiency of Mixture (1)

**Figure 1:**
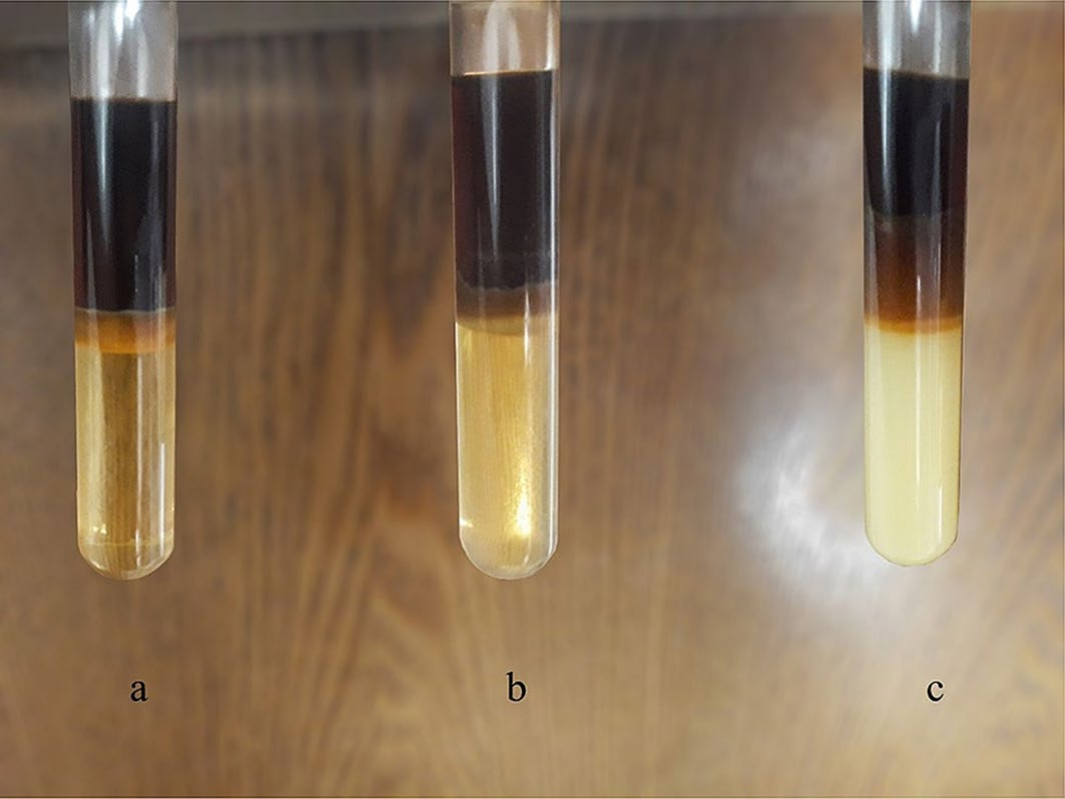
Phase separation results for the synthetic microemulsion system ($S\mu E$) formed with a) Mixtures (1), b) Mixture (2), and c) Mixture (3).

Here, the Mixture (1) performance on $S\mu E$ was evaluated; as shown in Fig. 1a, the oil and water phases separated spectacularly, forming the Winsor III. The TOC data in Table 1 indicates that the quantity of hydrocarbons in the aqueous phase is about 0.1 mg/L. Given the initial concentration of hydrocarbons in the system (0.5 mg/L based on TOC), nearly 80% of the surfactants are present in the bicontinuous region. For the sake of simplicity, we assumed that the excess phases are pure.

**Table 1:** TOC values and bottle test results for the $\mathrm{S\mu E}$ in the presence of all three mixtures of this research.

Mixture	TOC (mg/L)	Bottle test (%)
T80 + S20	0.1 ± 0.2^a^	90
K3 + Ak	0.15 ± 0.2	80
K6 + K3	0.33 ± 0.2	45

^a^TOC measurement uncertainty based on the TOC analyzer catalog.

However, the bottle test showed that Mixture (1) achieved approximately 90% of phase separation. To examine the stability of the system, we placed the vial at room temperature for two weeks. It is observed that the system did not experience any significant changes during this period, and the three-phase region's boundaries did not fluctuate widely. Therefore, the system achieved high stability using Mixture (1).

### The efficiency of Mixture (2)

Figure 1b shows a successful phase separation of $S\mu E$ by Mixture (2) since a distinguished bicontinuous phase is formed. As illustrated by the bottle test, the excess oil content is estimated to be around 80%, being less than that found in the presence of the Mixture (1). Furthermore, Table 1 reveals that the TOC value in the aqueous phase of $S\mu E$ is equivalent to 0.15 mg/L, which is higher than the previous case. So, these results prove the higher capability of Tween80 and Span 20 to treat this system.

As it can be seen, Mixture (1) could generate the excess aqueous phase with a brighter color compared to Mixture (2), justifying a higher tendency of Mixture (1) for separation. However, separation of synthetic microemulsion system in Mixture (2) was faster than in Mixture (1), and at similar conditions, the system needed about 9 h to reach the equilibrium. This difference is probably attributed to the surfactant's velocity when moving to the interface because the different structure of the surfactants considerably influences this process.

### The efficiency of Mixture (3)

As shown in Fig. 1c, Mixture (3) exhibited the minimum performance in $S\mu E$ and could not fulfill the overall goal. Although Mixture (3) achieved the desired HLD value, Table 1 clarifies that the separation process did not occur properly. Comparing these three surfactant mixtures, Mixture (1) could be the first choice for phase separation.

### HLD-NAC modeling of solubilization properties

In order to calculate solubilization volumes, values of length parameter (*L*), surface area (${a}_{i}$), and characteristic length ($\xi$) must be known. The procedure to fit NAC parameters developed by Acosta et al.^18^ is outlined in Supplementary Fig. S4, using the surfactants’ properties in Table 2.

**Table 2:** HLD-NAC parameters for the surfactants in the present study.

	Tween 80	Span 20	K3	K6	AK
Area per molecule, $a_i$ (Å^2^/molecule)	85	0	36	51	47
Surfactant parameter, *Cc*	− 3	3.5	2.8	− 0.5	− 0.2
*Mw*(g/mole)	1310	346	318	546	596
*v*_*s*_*/a*_*s*_ (Å)	25.8	*	16	17.8	21
Reference	^28^	^28^	This work	This work	This work

*This surfactant behaves as a lipophilic linker, with little participation in interface.

**Figure 2:**
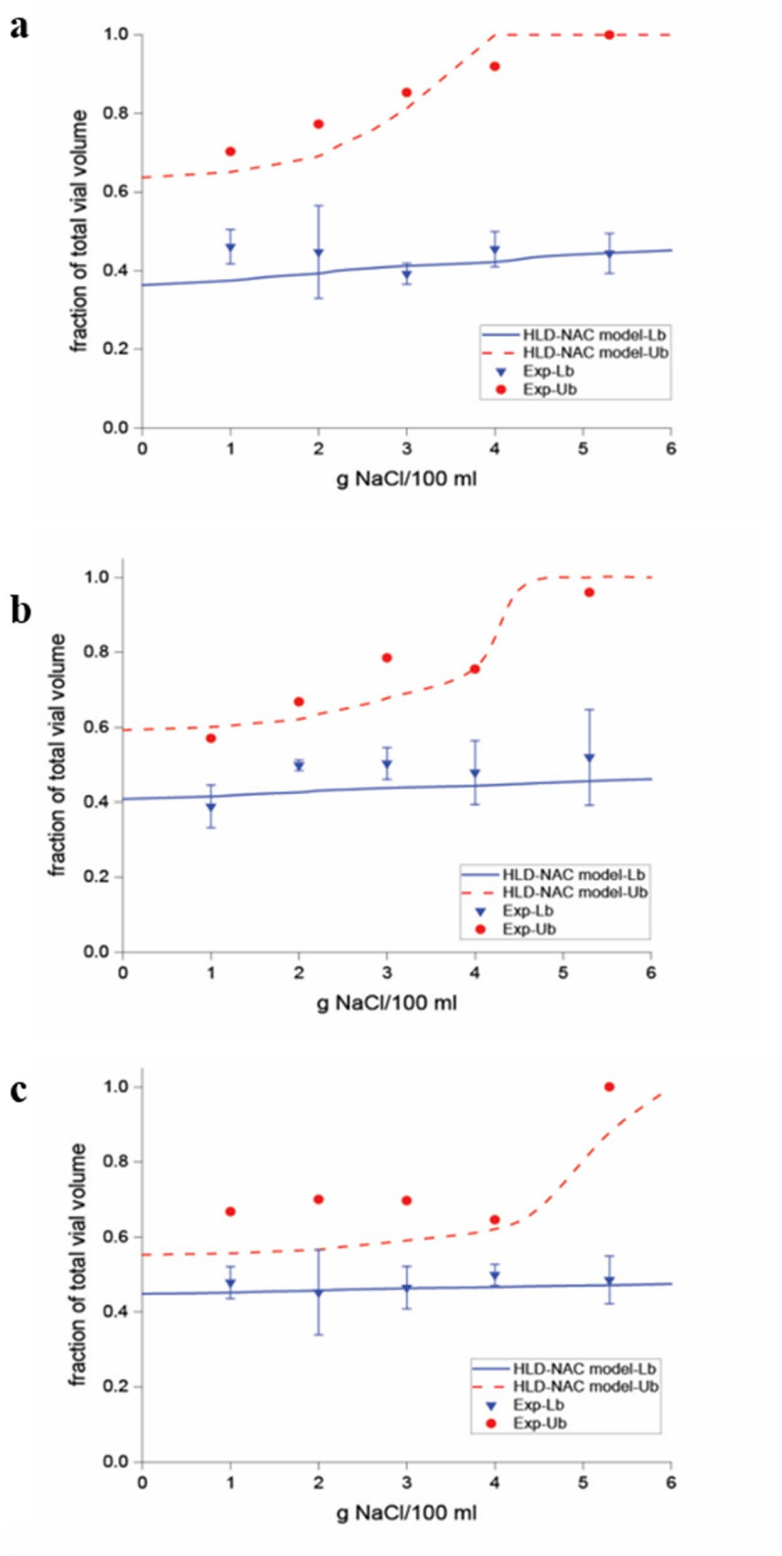
HLD-NAC volume fractions calculated with salinity scan (salinity between 1 and 5 g/100 mL at room temperature) for the synthetic microemulsion system ($S\mu E$) formed with (**a**) Mixture (1) (**b**) Mixture (2), and (**c**) Mixture (3). After minimizing the objective function, which is described in the supplementary file, using the experimental fractional volumes (circles and triangles) and those predicted by the HLD-NAC model (solid and dashed lines), *L* = 140 and $\xi$=528 for Mixture (1), *L* = 68 and $\xi$ =220 for Mixture (2), and *L* = 36 and $\xi$=104 for Mixture (3) were measured.

Supplementary Figure S5 shows the phase scan for the $\mathrm{S\mu E}$ system containing Mixture (1) from the salinity of 1 to 5 g/100 mL. The experimental equilibrated phase volume fractions of $\mathrm{S\mu E}$ for Mixture (1), (2), and (3) are shown in Fig. 2, along with the predicted phase volume fractions based on the HLD-NAC model. The best-fitted value for *L* and $\xi$ has been summarized for all three mixtures in Table 3. As can be seen, HLD-NAC predicts the phase volumes within an acceptable deviation. The surfactant parameters used in this study are reported here for the first time, except for Tween 80 and Span 20. According to Table 3, the value of predicted *L* for systems containing Span 20 is substantially large compared to the other surfactants. This large value is mainly related to the role of Span as a lipophilic linker which consequently increases the solubilization capacity in the microemulsion systems^31^. The presence of lipophilic linkers results in the ordered arrangement of oil molecules next to the surfactants’ hydrophobic part and the substantial upward trend of the characteristic length^32^.

### Performance of surfactant mixtures by the quality of the optimal formulation

Salager found that the power of HLD predictions in the three-phase region depends on the Winsor interactions or the affinity of the surfactants for both phases. He showed that the narrowest region of the middle-phases is the best condition to verify the HLD concept^33^. On the other hand, Acosta et al. stated that NAC parameters could express the three-phase region. Therefore, the characteristic length and the surfactant tail can evaluate the HLD at the phase transition point (Eqs. (4) and (5)). To the best of our knowledge, we connect these two distinct ideas for the first time in terms of demulsification performance. We found that, even though the HLD is zero, the formulation-related quality at optimum condition does not remain the same for all surfactants. Therefore, we are evaluating the performance of surfactant mixtures by quantifying the quality of the optimal formulation. The HLD values at the phase transitions obtained by NAC parameters ($\xi$, *L*) are listed in Table 3. These values enable us to determine the width of the bicontinuous range

**Table 3:** HLD transition values predicted by $\upxi$ and L for the synthetic microemulsion system containing Mixures (1), (2), and (3).

Mixture name	${\varvec{\xi}}$	*L*	HLD Transition
T80 + S20	528	140	$\pm$ 0.386
K3 + Ak	220	68	$\pm$ 0.506
K3 + K6	104	36	$\pm$ 0.633

.

It is evident that the smaller the ratio of $2L/\xi$, the narrower the transition phase width. Since the middle phase in the Mixture (1) is the narrowest one ($-$ 0.386 < Winsor III < 0.386), the HLD concept is more effective for this system and, this system forms Winsor III with higher efficiency. It is worth noting that this finding is in accordance with other results based on the shape of droplets (${L}_{d}/{R}_{d}$) and TOC. Hence, by quantifying the quality of optimal formulation using a simple salinity scan, a more effective surfactant to demulsify an emulsion is chosen among the candidate surfactants, which satisfies HLD = 0. This approach contributes to the improvement of a more sophisticated connection between demulsification and HLD-NAC.

### Performance of surfactant mixtures by the shape-based NAC model

As mentioned, calculating the droplets radii in the previous section are the key steps to obtain oil and water volumes in the $S\mu E$. The assumption of the existence of spherical droplets might not be accurate in the phase transition regions. Acosta et al. modified the physical interpretations of droplets and developed the following equations. They also provided a significant implication for understanding how the simulated shape of droplets changes by varying HLD using the following equations^34^:

6 $${H}_{n}^{^{\prime}}=\frac{{H}_{n}}{2}=\frac{2}{{L}_{d}+{2R}_{d}} + \left(\frac{{L}_{d}}{{R}_{d}}\right)\left(\frac{1}{{2L}_{d}+{4R}_{d}}\right)$$

7 $${H}_{a}=\frac{2{L}_{d}+4{R}_{d}}{{3L}_{d}{R}_{d}+{4R}_{d}^{2}}$$

The simulated shape of droplets in the microemulsion systems consists of a cylindrical neck region of length (${L}_{d}$) and hemispherical end caps of radius (${R}_{d}$)^35^. When approaching the three-phase region, micelles' shapes change from a sphere (${L}_{d}$=0) to rods (${L}_{d}\gg {R}_{d}$), highlighting that the state of the system shifts into bicontinuous channels. Here, for the first time, this approach is applied to evaluate the separation efficiency of each surfactant mixture by comparing the ratio of ${L}_{d}/{R}_{d}$.

**Figure 3:**
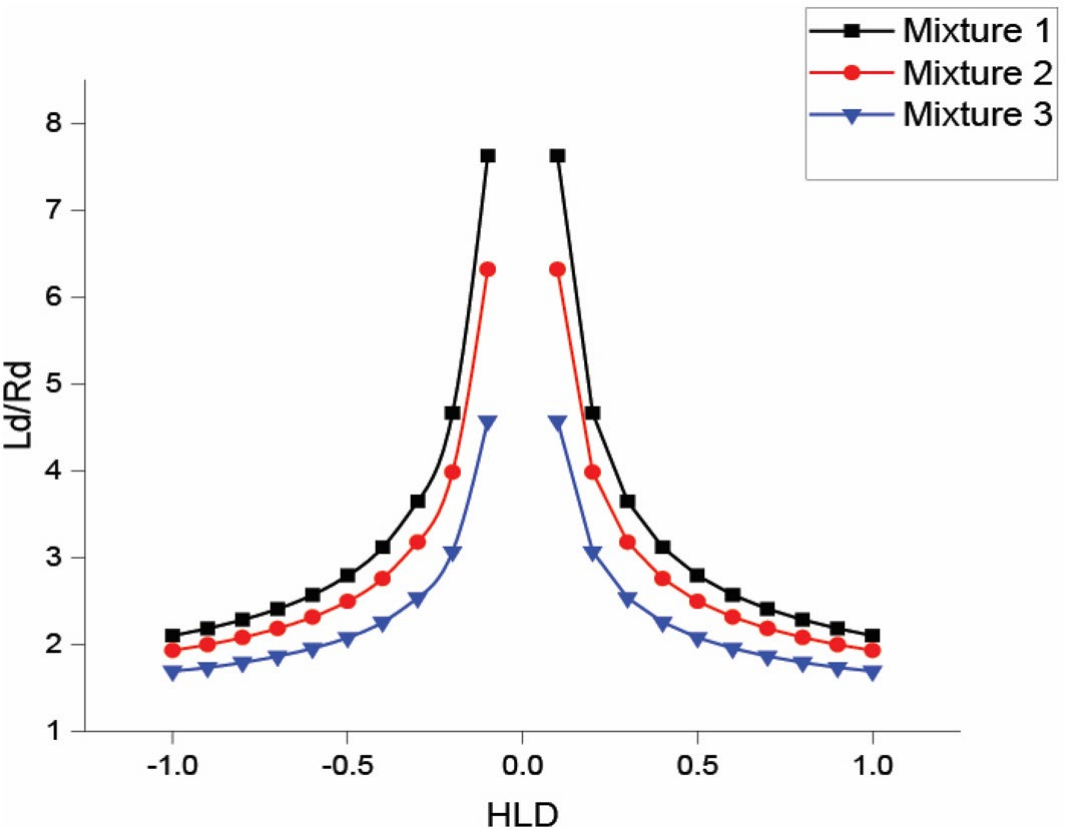
The ratio of cylindrical length to radius of microemulsion droplets (${L}_{d}/{R}_{d}$) in the synthetic microemulsion system for all three mixtures predicted via the HLD-NAC model.

Supplementary Figure S6 shows *R*_*d*_ and *L*_*d*_ for all the mixtures in this study. As shown in Fig. 3, the ratio of ${L}_{d}/{R}_{d}$ for Mixture (1) is the highest. This implies that the system in the presence of this mixture can produce such elongated structures whose shapes look more like a network of bicontinuous channels than the other mixtures. In other words, the spherical droplets in a system with a high ${L}_{d}/{R}_{d}$ have more tendency to be converted to a bicontinuous system.

The predicted trends are consistent with the previous results in TOC analysis. Hence, ${L}_{d}/{R}_{d}$ is a valuable indicator for detecting the system’s entry into the transition phase region. Undoubtedly, more research is needed before this approach can be applied to more complex systems. For instance, salt concentration effects on the micelles’ core solubilization may influence the whole system, which is out of the scope of this study^19,34^.

**Figure 4:**
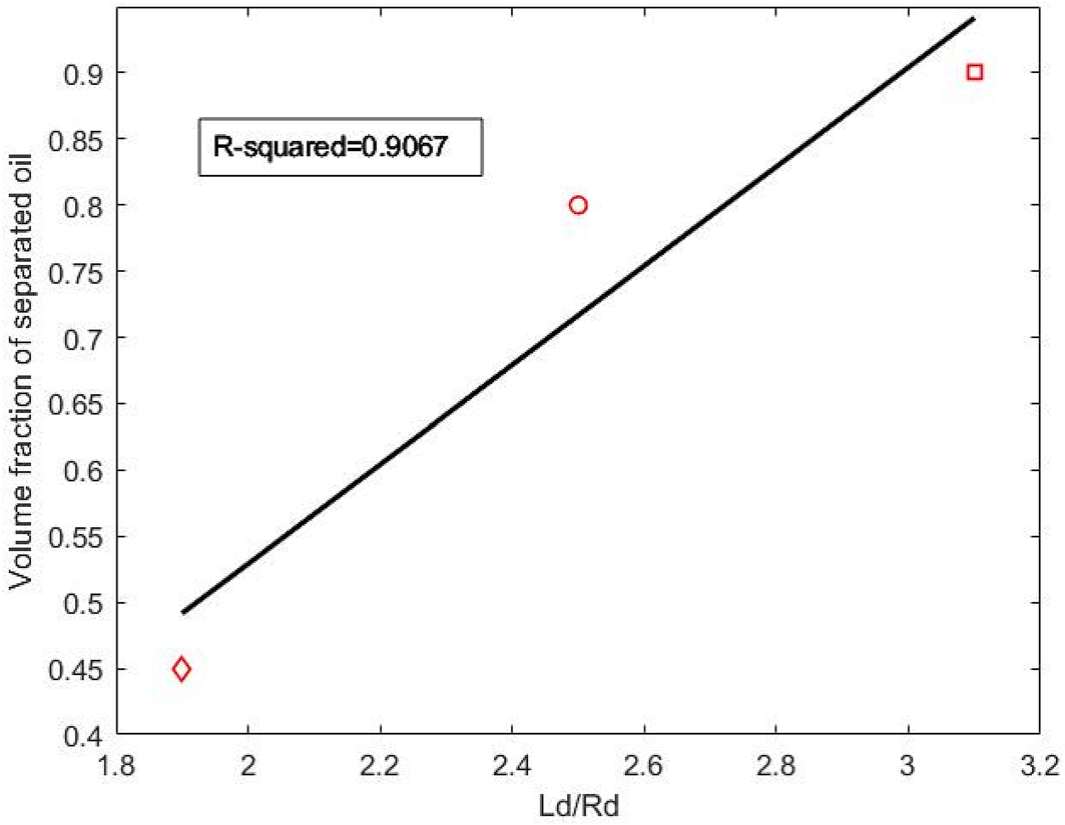
Bottle test results (volume fraction of separated oil) vs. (${\mathrm{L}}_{\mathrm{d}}/{\mathrm{R}}_{\mathrm{d}}$) at phase transition points in the synthetic microemulsion system for Mixture (1) (square), Mixture (2) (circle), and Mixture (3) (diamond).

Furthermore, a meaningful connection between HLD-NAC geometrical sizes of non-spherical droplets and demulsification behavior was found. It is hypothesized that a direct relationship exists between the volume fraction of separated oil in bottle test results and ${L}_{d}/{R}_{d}$ values at the phase transition point. Since ${L}_{d}/{R}_{d}$ varies as a function of HLD, the values of ${L}_{d}/{R}_{d}$ at phase transition points correspond to the HLD transition values. Figure 4 shows the plot of volume fraction of separated oil for $S\mu E$ systems containing all three surfactant mixtures versus the ${L}_{d}/{R}_{d}$ values of these systems at the phase transition point. Although further experiments are needed to confirm this hypothesis, correlating our experimental data verifies it with a high value of R-squared. It can be concluded that each emulsion system with higher ${L}_{d}/{R}_{d}$ at phase transition point performs better in terms of demulsification.

### Performance of surfactant mixtures in the presence of alcohols in the ${{S}}{{\mu}}{{E}}$ systems

In this research, sec-butanol was used to determine its effect on phase separation. According to the references, sec-butanol is neutral alcohol that accelerates the rate of separation^36^. The effect of sec-butanol on Mixture (1) in the $S\mu E$ system is shown in Supplementary Fig. S7. Measuring the phase separation time, which is about 12 h, we noticed a shorter equilibrium time by injecting the alcohol than without alcohol (72 h). Besides, sec-butanol causes minor deviation of the upper and lower boundaries of the middle phase in the $S\mu E$ system compared with the same system without sec-butanol. This behavior confirms its less significant contribution to the system's performance. Adding alcohol, we observe high turbidity in the system as a negative impact, which has an adverse effect on the demulsification process. In general, its advantages outweigh the negative effect on this system. In contrast, according to Supplementary Fig. S7, sec-butanol leads to poor performance in phase separation for Mixtures 2 and 3. As it was stated by Salager, in general, injecting the alcohol may affect all the formulation variables as it may partition in both oil and the aqueous phase. It seems that a non-ideal behavior of alcohol-surfactants in Mixtures 2 and 3 disturbs the balance in the SOW systems^12^.

### The efficiency of surfactant mixtures on demulsification process in IE systems

**Figure 5:**
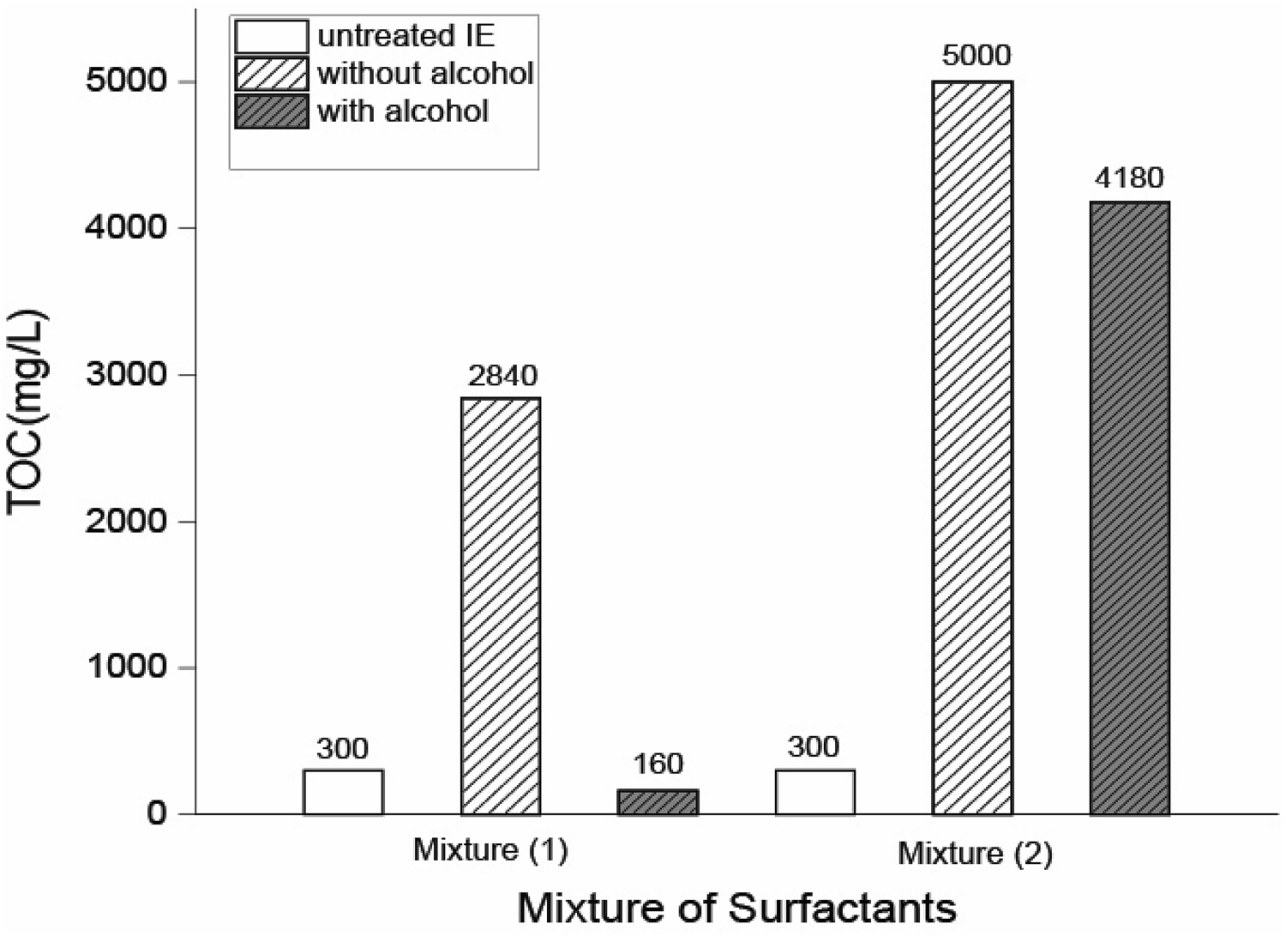
The comparison between TOC results of industrial wastewater system (*IE)* containing Mixture (1) and Mixture (2) before and after injecting sec-butanol. TOC measurement uncertainty is about 50 mg/L based on the TOC analyzer catalog.

Figure 5 presents the TOC results of Mixture (1) and Mixture (2) in the *IE* system with and without alcohol. TOC of the industrial wastewater without any treatment is 300 mg/L. After the demulsifier injection—without any alcohol—TOC for Mixture (1) and (2) rises to 2840 mg/L and 5000 mg/L, respectively. This may be related to the organic nature of the demulsifiers, demonstrating that HLD prediction was not effective in the treatment of this system, and the phase separation was not carried out adequately. Of course, this was not unexpected, given that HLD has been designed for systems with water in oil ratios of 1:1.

**Figure 6:**
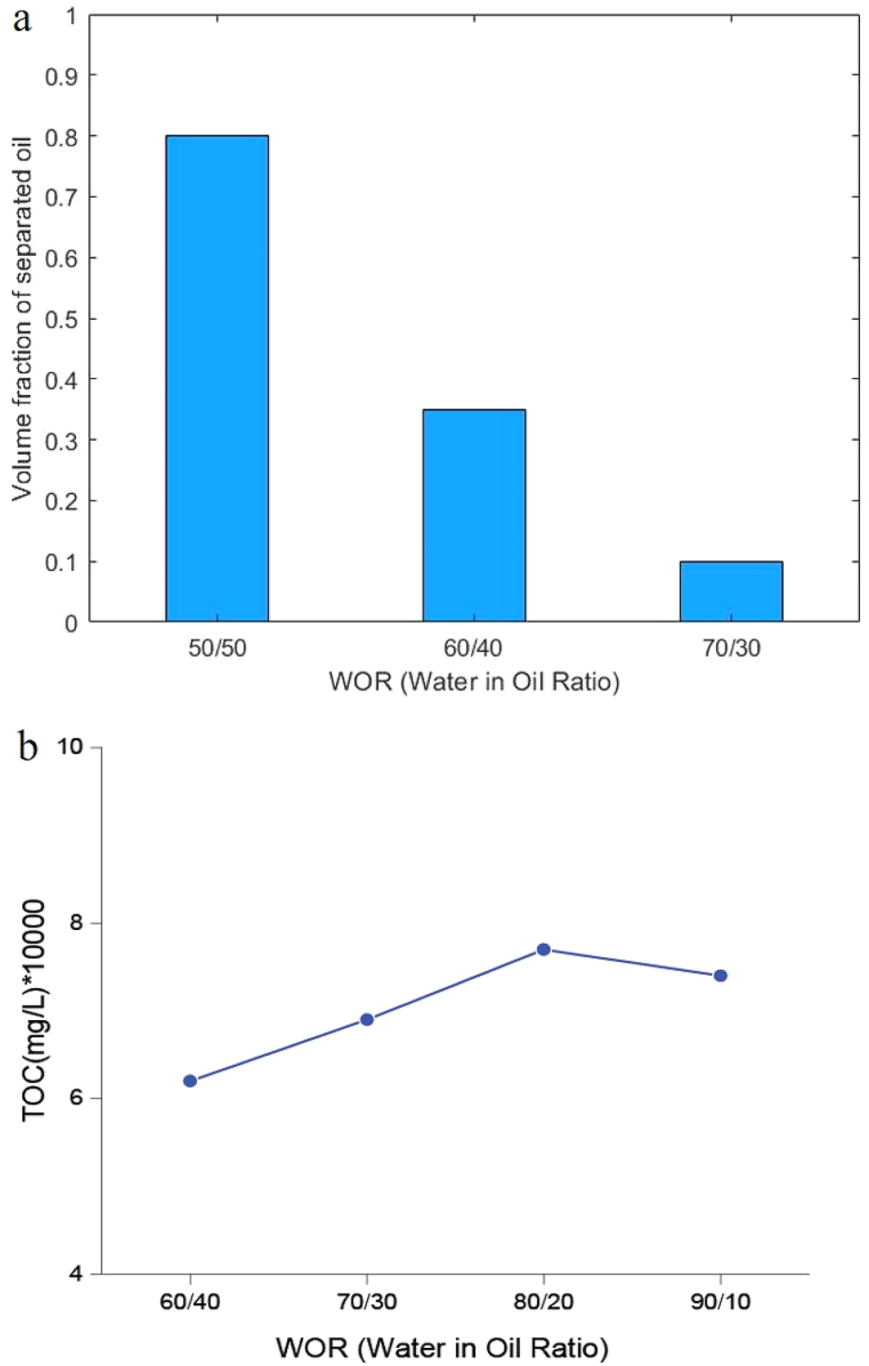
Bottle test results (volume fraction of separated oil) (**a**) and TOC of the separated water phase (**b**) for tubes of $S\mu E$ systems with different water in oil ratios. TOC measurement uncertainty is about 50 mg/L based on the TOC analyzer catalog.

When dealing with real wastewater, the volumes of water are substantially higher. Therefore, the hydrophilic-lipophilic nature of the system alters significantly, and the amounts of consumed surfactant and the *Cc* value required to reach HLD = 0 for this real wastewater are not in accordance with the synthesized emulsion with WOR = 1. To inspect the effect of WOR on the separation efficiency, we prepared the $S\mu E$ tubes similar to the previous section but with different water in oil ratios, ranging from 60/40 to 90/10. Due to the fast phase separation in using Mixture (2), we chose this mixture to evaluate the demulsification efficiency in the *IE* system. The findings were illustrated in Supplementary Fig. S8. As demonstrated in Fig. 6a, aside from the 50/50 ratio (WOR = 1), which exhibited 80% separation, 60/40 and 70/30 ratios showed considerable separation performance of 35% and 10%, respectively. In other words, results indicate that the predictive power of HLD has lowered steeply with the increase in the water in oil ratio. Besides, the TOC results in Fig. 6b confirmed that the surfactant parameter designed by HLD is not suitable for the WOR far from 1.

Generally speaking, poor solubility of surfactant in the continuous phase creates significant obstacles for SOW systems formulation. In order to overcome this issue, the HLD-NAC approach is proposed, and the fish diagram can provide the information to appropriately decide what changes are needed to accomplish the separation target. When the water volume in the SOW system is considerably higher than oil volume, similar to wastewaters, there is not enough space at the interfaces to occupy all the surfactants; thus, the excess surfactants have to disperse in the water phase. If the surfactants are not hydrophilic enough, the system shifts to a more hydrophobic state, and the system’s balance will be upset. As a result, the wastewater requires a more hydrophilic (more negative *Cc*) surfactant to reach the optimal value than the systems with the same conditions at WOR equal to 1 (Supplementary Fig. S9).

### Performance of surfactant mixtures with alcohols in IE systems

In most literature, sec-butanol is considered neutral alcohol, and its effect was also reported in our experiments at WOR = 1. Nevertheless, partitioning of the alcohol in oil and water phases strongly depends on other system variables, especially the water in oil ratios^37^. Besides, sec-butanol is water-soluble, and its constant(*m*_*a*_) in HLD equation (*f(a)*) is slightly negative^12^. As mentioned in the previous section, with the higher ratios of water to oil, the balance of the SOW system becomes more hydrophobic. This behavior can be restored by exerting a hydrophilic effect—the presence of sec-butanol here—on the system. Therefore, the effect of sec-butanol in WOR 1 is required to be examined. In Supplementary Fig. S10, sec-butanol behaved surprisingly in Mixture (1) in terms of separation. The TOC of the system presented in Fig. 5 demonstrates that after injecting sec-butanol in Mixture (1), the TOC dropped from 300 mg/L—the initial concentration of oily wastewater—to 160 mg/L. It is well illustrated in Supplementary Fig. S10 that a layer of oil is separated at the top of the tube and sec-butanol plays the role of a co-solvent in the system, which provides a sufficient hydrophilic condition for the demulsification process. As a result, having a compatible demulsifier that can be obtained through a proper solvent is a critical stage in selecting a proper demulsifier.

However, despite the advantages of using the alcohol for *IE* system with Mixture (1), sec-butanol could not still be successful for the same system with Mixture (2) since TOC increased to 4180 mg/L as shown in Fig. 5. Adding sec-butanol to Mixture (2) is a better option than the same condition without sec-butanol, although this considerable difference cannot satisfy our demulsification target. Most probably, this is the result of the poor solubility of the mixture in sec-butanol. In fact, sec-butanol is not an appropriate solvent to tackle the solubility issue of industrial wastewater with Mixture (2). Besides, the solubility data of the commercial surfactants was not available. This investigation on the relation between HLD and WOR can provide some evidence that *Cc* obtained by HLD can only act as an initial guess for systems with WOR $\ne$ 1.

### The effect of surfactant concentration on ${{S}}{{\mu}}{{E}}$ systems

The surfactant concentration should not be less than the concentration needed to achieve the microemulsion formulation of Winsor III. Generally, the concentration values between 5 and 10 wt% are selected. This section, it is aimed to understand the effect of surfactant concentration on the separation efficiency in $S\mu E$ system*s*. An investigation was performed on Mixture (1), and surfactant concentrations of 0.5, 2, and 4 were prepared based on weight percent. According to Supplementary Fig. S11, the maximum separation occurred in Cs = 4, and as the concentration decreased, the amount of separation was strongly dropped. As stated in the review paper by Salager et al., when a mixture of surfactants is used, the *Cc* parameter corresponding to the optimum formulation is usually not fixed and will change by surfactant concentration. It was also noted that this discrepancy is more critical when the surfactant concentration is low enough near the critical microemulsion concentration (*C*_*m*_). In this experiment, Mixture (1)—which is the mixture of two commercial surfactants—is used. Therefore, we expect discrepancies in *Cc* parameter and change in separation performance with an alteration of surfactant concentration. Since *Cc* parameters of commercial surfactants during the salinity scan are measured at five weight percent of surfactants, we can conclude that the more we deviate from this measured surfactant concentration, the lower separation performance is observed. However, this deviation is more critical at lower concentrations, for example, near 0.5 wt%, since it is nearer to *C*_*m*_.

## Conclusions

HLD is currently a suitable model to find the state of different kinds of microemulsion and emulsion systems and shows a reasonable accuracy at oil in water ratios near one. This work implements a stepwise procedure to measure HLD parameters (*Cc* and *EACN*) for both synthesized microemulsion and industrial wastewater systems and obtain NAC parameters (*L*, $\xi$) by salinity scan for the synthetic microemulsion system. The phase separation of two systems of interest is evaluated using TOC and bottle tests. This work clarifies that HLD can measure the parameters of commercial surfactants and highlights the importance of the NAC framework when the optimum experimental condition does not correspond to HLD = 0. Using the HLD-NAC for $S\mu E$ systems, parameters of simulated shape of droplets are obtained, and higher separation efficiency of Mixture (1) is verified. In other words, more effectiveness in phase separation is accompanied by entering the Winsor III region, which can be demonstrated by a higher value of ${L}_{d}/{R}_{d}$ and higher quality of optimum formulation. Furthermore, the effect of sec-butanol on phase separation is investigated in both systems, and it is found that this alcohol—probably in the role of co-solvent—can produce desirable changes in real systems to achieve demulsification.

## Supplementary Information

Supplementary Information.
