# Supplementary material for: Design of industrial wastewater demulsifier by HLD-NAC model
Source: Sci Rep. 2021 Aug 9;11:16111. doi: 10.1038/s41598-021-95485-7 (PMC8352873; doi:10.1038/s41598-021-95485-7)
Supplement: Supplementary file 1 — Supplementary Information. [file 41598_2021_95485_MOESM1_ESM.pdf]

Supplementary Information File *for the article:*

Design of Industrial Wastewater Demulsifier by HLD-NAC Model

Hassan Ghasemi[1], Fatemeh Eslami[2*]

[1]Department of Chemical Engineering, Tarbiat Modares University, Tehran, Iran

[2]Department of Chemical Engineering, Tarbiat Modares University, Tehran, Iran

Corresponding Author:

Fatemeh Eslami

Email: f_eslami@modares.ac.ir

Content:

This file includes 9 pages (S2–S10), not including the cover page, eleven figures (Figures S.1–S.11), and two tables (Tables S.1 and S.2). The following subjects are covered in the file:

Determination of EACN and Cc through salinity scan

Fitting the HLD-NAC parameters

L_d and R_d values predicted by HLD-NAC

Role of alcohol in the phase separation of synthetic microemulsion system

Effect of WOR on the separation efficiency for tubes of SμE systems, ranging from 60/40 to 90/10.

Examination of WOR ratio on the balance of the SOW system through fish-diagram

Role of alcohol in the phase separation of industrial emulsion

Role of demulsifier concentration in the phase separation of synthetic microemulsion system

Determination of EACN and Cc through salinity scan

It is generally accepted that the salinity scan is commonly used to calculate *EACN* and *Cc* parameters. There is a series of test tubes in the salinity scan method where a fixed amount—which is typically 3-5 wt.%— of a surfactant or a mixture of surfactants with known *Cc* parameters were prepared in equal volumes of oil and water. After changing the salinity of tubes as the variable parameter, the tubes were shaken gently 20 times by hand and left to equilibrate at room temperature for at least 48h; a longer time may be required for other mixtures of surfactants because of the difference in interfacial rigidity of the surfactant film.

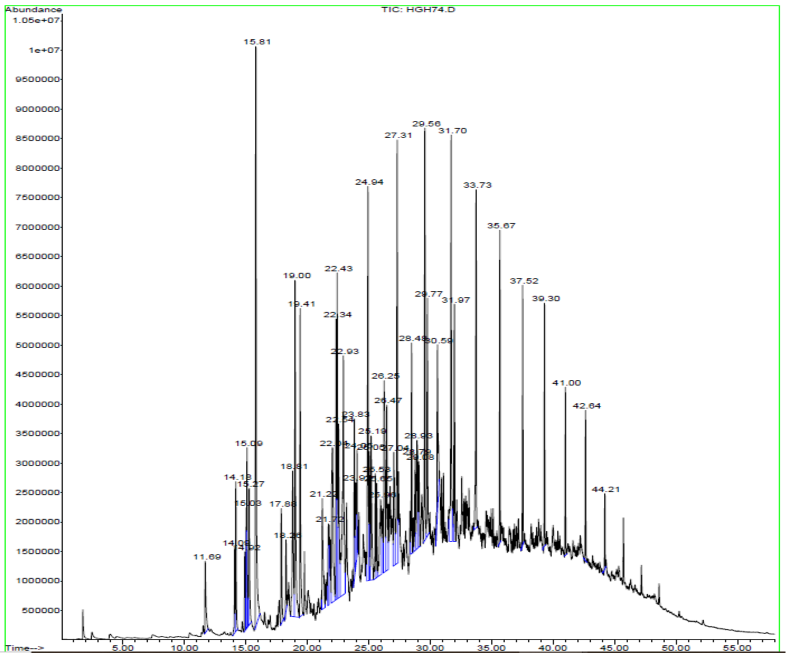

Fig. S1. GC/MS analysis for the contaminant oil

In order to do the salinity scan to measure *EACN* at 25°C, based on the reported guess for *EACN*, the range of 1 to 5 for salinity and *Cc* =−0.4 are chosen as the appropriate scan scope for measurement of *EACN*. SDS and AOT formed a mixture of surfactants to produce this value based on the information presented in Table S1. Assigning the mentioned salinity range in Eq. (1), the calculated *EACN* is shown in Table S2.

Table S1

Properties of SDS and AOT in EACN measurement

Surfactant	Molecular weight (g/mole)	*Cc*
**SDS**	288.37	-2.3
**AOT**	444.56	2.55

Table S2

Salinity range values for calculating accurate EACN

Salinity (g/100 mL)	*EACN*
**1**	−2.3
**2**	1.7
**3**	4.1
**4**	5.8
**5**	7.1

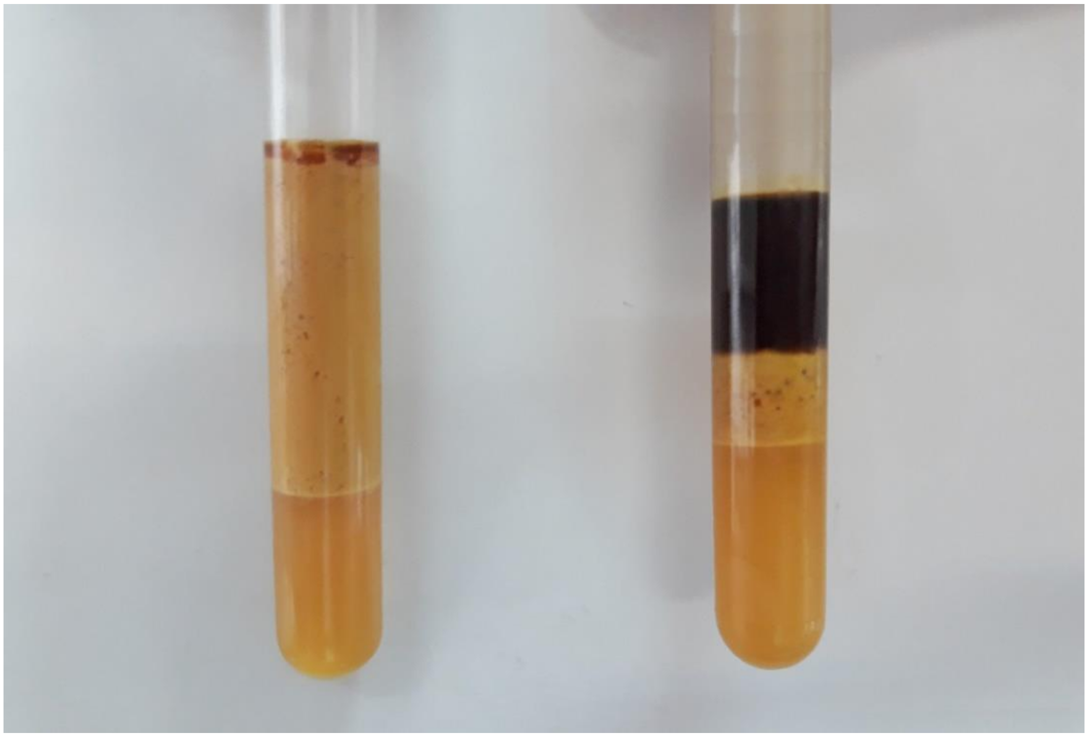

Fig. S2. Performing Salinity scan to obtain *EACN* value of contaminant oil. It is performed in the microemulsion systems formed with a mixture of AOT & SDS as a mixture of surfactants at 25 °C. From left to right, the concentration of NaCl as salt is 4 and 2 g/100mL, and Winsor III is only observed in the tube with *S*=2, resulting in *EACN*=1.7.

Similarly, the salinity scan is used to measure *Cc* of surfactant mixtures of the work. Fig. S3 presents the measured through salinity scan.

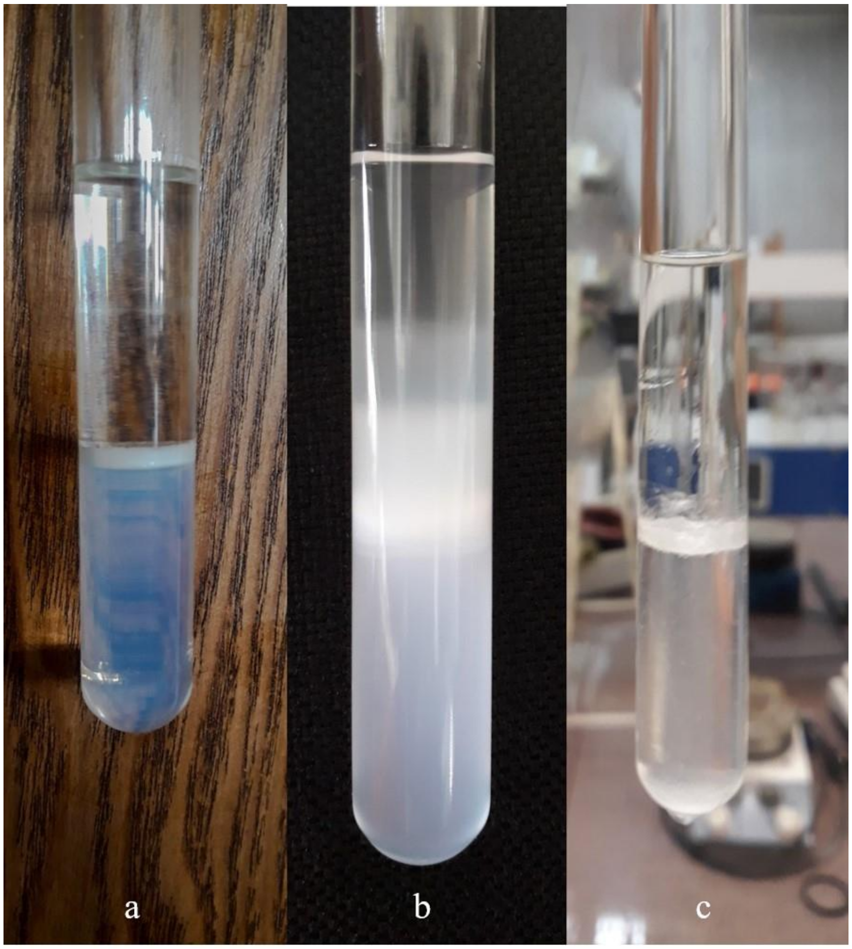

Fig. S3. Salinity scan results for determining the *Cc* of surfactant a) AK, b) K6, c) K3

Fitting the HLD-NAC parameters

Fig. S4 illustrates the flowchart for calculating the necessary input data for solubilization capacities and estimation of the physical characteristics of the system via HLD-NAC. This procedure follows the similar algorithms introduced by Ghayour and Acosta[1], predicting values of length parameter (L) and characteristic length (ξ). By conducting the salinity scan for three mixtures of surfactants in $S\mu E$ and minimizing the objective function the values of L and ξ are determined.

$$\text{Objective Function} = \sqrt{\Sigma\left(\left(Ub_{cal} - Ub_{exp}\right)^2 + \left(Lb_{cal} - Lb_{exp}\right)^2\right)}$$

Ub is the boundary of upper and middle phases while Lb is the boundary of the lower and middle phases. It can be seen that as salinity increases, the system experiences gradual phase transition from Winsor III to II as Fig. S5 shows the changes in phase volume fractions for Mixture (1).

1 Ghayour, A. & Acosta, E. Supplementary information file for the article : Characterizing the oil-like and surfactant like behavior of polar oils. 1–10.

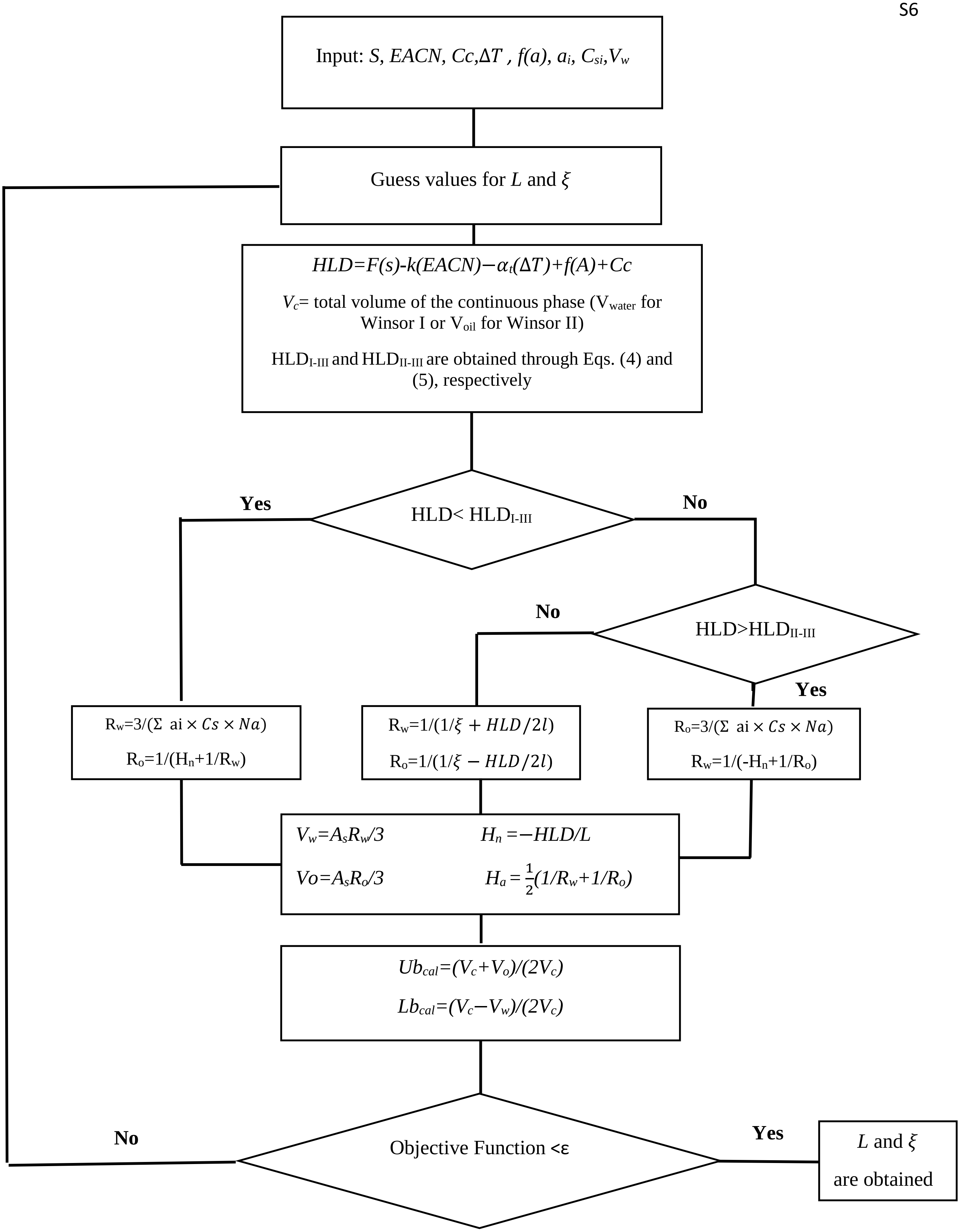

Fig. S4. Flowchart of HLD-NAC for optimizing L and ξ.

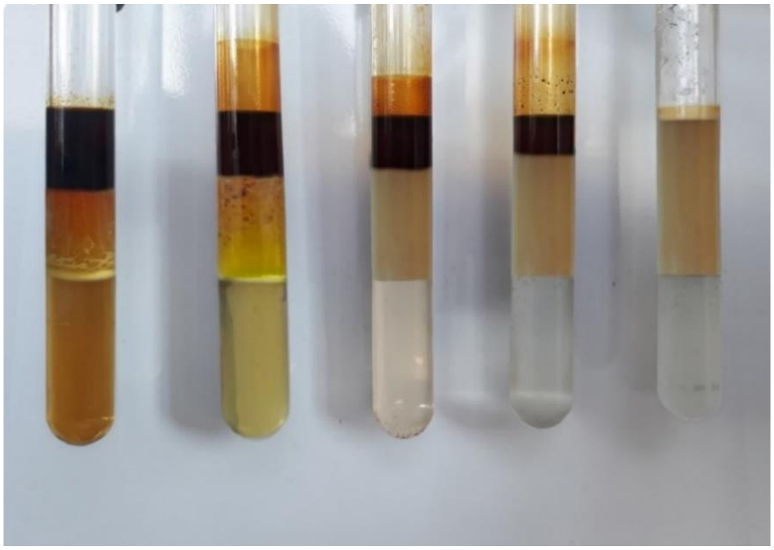

Fig. S5. Phase scan for the synthetic microemulsion system ($S\mu E$) containing Mixture (1) from the salinity of 1 (left) to 5 g/100 mL. As salinity increases, the system experiences a gradual phase transition from Winsor III to II. All experimental data were replicated three times.

L_d and R_d values predicted by HLD-NAC

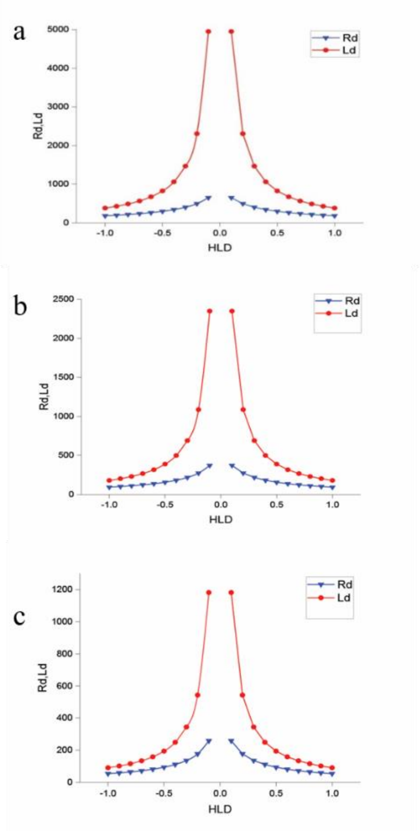

Fig. S6. L_d and R_d values of droplets in the $S\mu E$ for a) Mixture (1), b) Mixture (2), and c) Mixture (3) predicted by HLD-NAC. Data are illustrated at different HLD values.

Role of alcohol in the phase separation of synthetic microemulsion system

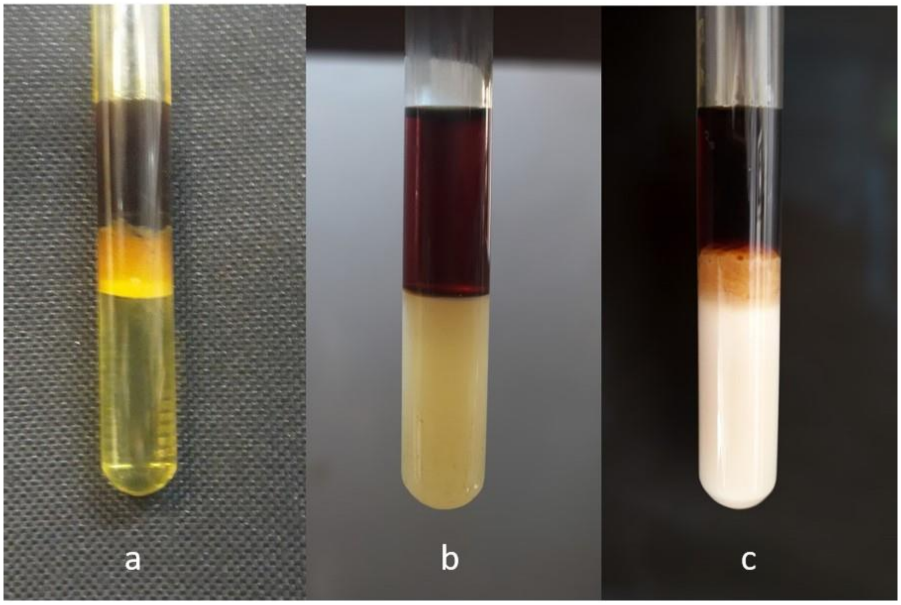

Fig. S7. Effect of sec-butanol on the synthetic microemulsion system formed with a) Mixture (1), b) Mixture (2), and c) Mixture (3)

Effect of WOR on the separation efficiency for tubes of $S\mu E$ systems, ranging from 60/40 to 90/10.

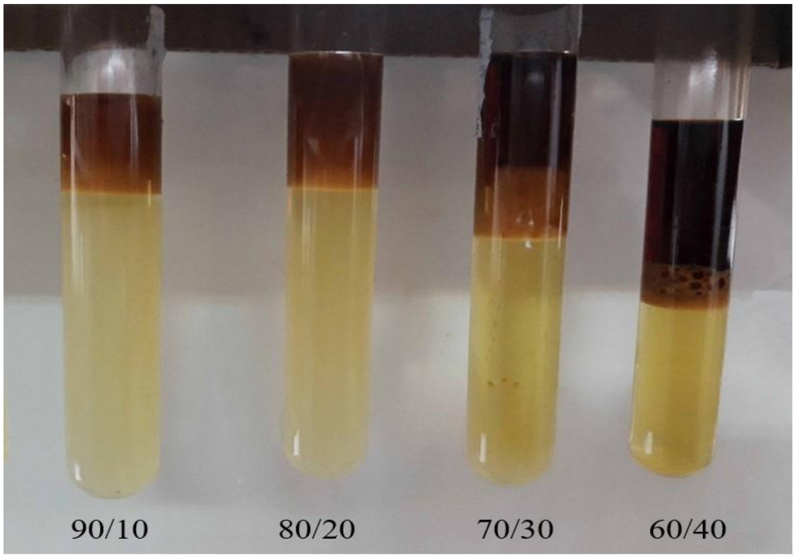

Fig. S8. Tubes of $S\mu E$ systems formed with Mixture (2) at different water in oil ratios. WOR varies from 60/40 to 90/10

Examination of WOR ratio on the balance of the SOW system through fish-diagram

Usually, the fish diagram is plotted for the systems with water in oil ratios close to 1 with the format of Y versus the surfactant concentration. Y can be any property of the system such as T, *Cc*, and *EACN*, while it is usually chosen to be temperature and the X point of the diagram corresponds to PIT. For each WOR, the fishtail corresponding to HLD=0 occurs at a specific surfactant concentration. Understanding how the fishtail change by WOR can aid in selecting suitable demulsifiers to achieve HLD=0. In Fig. S9, the square and star dots denote the fishtail (X-point) position which corresponds to HLD=0 for different water in oil ratios. It can be seen that by increasing the WOR, Cc moves to smaller values. It suggests that for higher WOR ratios, the more appropriate surfactant for satisfying HLD=0 is likely to be more hydrophilic.

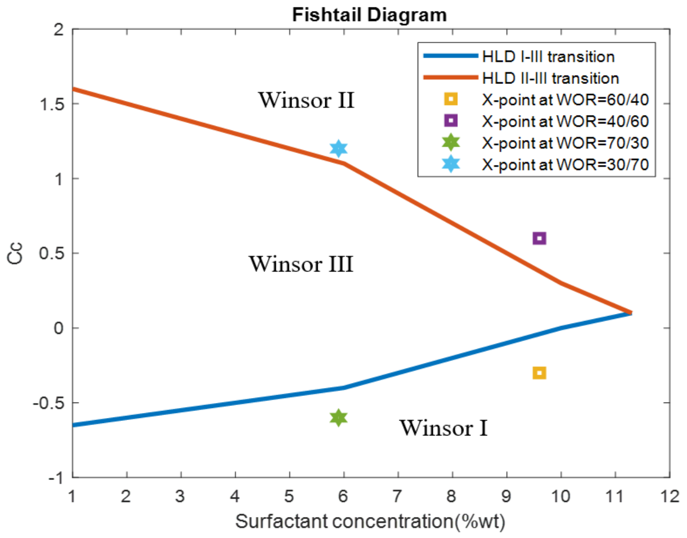

Fig. S9. *Cc*-Fishtail diagram for a SOW system with S=2, *EACN*=6 at room temperature and WOR=1 reproduced from Abbott interactive website[2]. Also, X-points position at different water in oil ratios is shown. With the increase and decrease in WOR of the system, *Cc* values decrease and increase, respectively.

2 Abbott, S. Fishtail Diagrams. Prof Steven Abbott https://www.stevenabbott.co.uk/practical-surfactants/fishtail.php (2015).

Role of alcohol in the phase separation of industrial emulsion

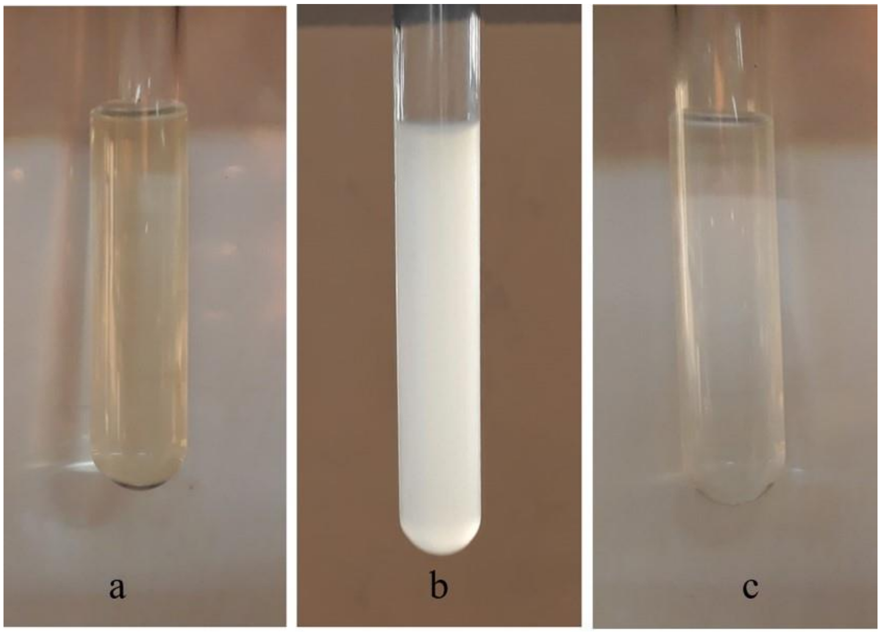

Fig. S10. Demulsification results of Mixture (1) in the industrial wastewater system (IE) a) untreated b) without sec-butanol c) with sec-butanol

Role of demulsifier concentration in the phase separation of synthetic microemulsion system

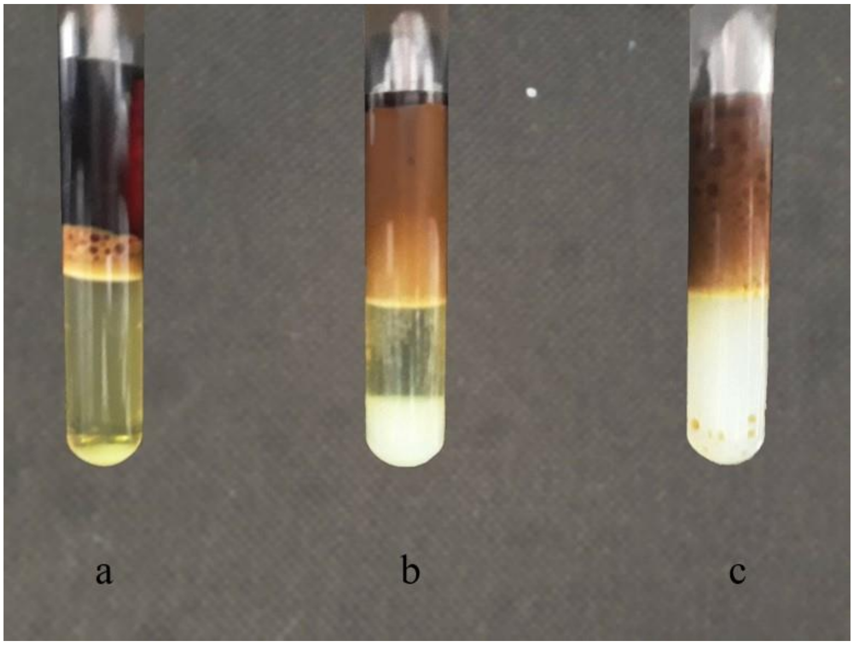

Fig. S11. Effect of demulsifier concentration on phase behavior of $S\mu E$ at concentrations a) 4 b) 2, and c) 0.5 wt.%.
